# 4D Flow Assessment of Vorticity in Right Ventricular Diastolic Dysfunction

**DOI:** 10.3390/bioengineering4020030

**Published:** 2017-04-05

**Authors:** James R. Browning, Jean R. Hertzberg, Joyce D. Schroeder, Brett E. Fenster

**Affiliations:** 1 Northeastern University, Department of Mechanical Engineering, Boston, MA 02115, USA; 2 University of Colorado, Department of Mechanical Engineering, Boulder, CO 80309, USA; jean.hertzberg@colorado.edu; 3 University of Utah School of Medicine, Department of Radiology, Salt Lake City, UT 84132, USA; joyce.schroeder@hsc.utah.edu; 4 National Jewish Health, Division of Cardiology, Denver, CO 80206, USA; fensterb@njhealth.org

**Keywords:** 4D flow cardiac MRI, diastolic dysfunction, pulmonary arterial hypertension, right ventricle, vortex

## Abstract

Diastolic dysfunction, a leading cause of heart failure in the US, is a complex pathology which manifests morphological and hemodynamic changes in the heart and circulatory system. Recent advances in time-resolved phase-contrast cardiac magnetic resonance imaging (4D Flow) have allowed for characterization of blood flow in the right ventricle (RV) and right atrium (RA), including calculation of vorticity and qualitative visual assessment of coherent flow patterns. We hypothesize that right ventricular diastolic dysfunction (RVDD) is associated with changes in vorticity and right heart blood flow. This paper presents background on RVDD, and 4D Flow tools and techniques used for quantitative and qualitative analysis of cardiac flows in the normal and disease states. In this study, 20 patients with RVDD and 14 controls underwent cardiac 4D Flow and echocardiography. A method for determining the time-step for peak early diastole using 4D Flow data is described. Spatially integrated early diastolic vorticity was extracted from the RV, RA, and combined RV/RA regions of each subject using a range of vorticity thresholding and scaling methods. Statistically significant differences in vorticity were found in the RA and combined RA/RV in RVDD subjects compared to controls when vorticity vectors were both thresholded and scaled by cardiac index.

## 1. Introduction

Three-dimensional blood flow characteristics in the right heart (RH), including wall generated shear and coherent fluid structures, remain relatively unstudied and may have functional significance in the pathology of cardiac disease. Due to its relatively clear role in heart disease, flow characteristics of the left ventricle in the normal and pathologic state have been well studied, including the diastolic vortex ring which forms downstream of the mitral valve (MV) and whose properties have been shown to correlate with markers of left ventricle (LV) diastolic dysfunction [1,2,3]. Diastolic vortex rings have also been observed in the right ventricle (RV) but their pathological significance has been less well characterized [4,5,6,7,8]. Right atrial (RA) flow patterns have received even less attention but 4D Flow studies have described overall characteristics in normal subjects. For example, Kilner et al. showed that a right-handed helix originates in the convergence of inferior and superior vena cava streams during systole [9,10]. During diastole, the blood entrained in this helix flows through the tricuspid valve (TV) and into the right RV, where flow recirculating beneath the leaflets may form a partial vortex ring. Studies have found that these RV vortex structures may be altered in canine models of PH as well as human subjects with repaired tetralogy of Fallot and Fontan circulations [11,12,13,14].

Because vorticity is a derivative quantity of the velocity field, we hypothesize that it is sensitive to subtle changes in cardiac flows concurrent with cardiovascular pathologies. Vorticity is often present in coherent flow structures such as helices, ring and line vortices, as well as in shear flows and boundary layers [15]. Vorticity may offer novel and robust diastolic flow characterization with at least incremental value compared to existing echocardiographic techniques. Using time-averaged phase-velocity encoded acquisition in the X, Y, and Z planes, 4D Flow can generate high-fidelity spatial and temporal renderings of the velocity field and offer a straight-forward method for quantifying vorticity.

Given the aforementioned relationship between LV vortex structures and cardiac pathologies, we hypothesize that similar correlations exist between right heart vorticity and right ventricular diastolic function. The adaptive changes that result from chronic pressure overload in pulmonary arterial hypertension (PAH) lead to myocardial hypertrophy, stiffening, and right ventricular diastolic dysfunction (RVDD) [16], and are thus likely to affect the vorticity field. Additionally, a growing body of evidence has identified RVDD as an important prognostic indicator for PAH [17,18]. We have previously shown that 4D Flow-derived RH vorticity correlates with echocardiographic indices of RVDD in a small cohort of subjects [19]. Using 4D Flow for the study of RH hemodynamics may provide clinical tools for the diagnosis of cardiac pathologies as well as further understanding of the mechanics of RVDD and PAH. Metrics derived from vorticity analysis in particular are expected to provide a sensitive noninvasive diagnostic tool, whether based on 4D Flow measurements or echocardiographic data. As a first step, this prospective study compares controls to PAH subjects with RVDD, focusing on vorticity in the RA, RV, and RH (RA + RV) volumes during early diastole. Our goals are exploratory in nature: to develop tools for examination of the basic fluid dynamics which may then lead to the selection of a metric for disease progression. Total vorticity is used as a potential proof-of-concept metric which will provide guidance for future analyses using more sophisticated automated coherent structure analysis techniques such as Q-criterion, Lambda_2_, helical decomposition, and Lagrangian coherent structures [20]. The relationship of the metrics to the physics of disease progression as well as development of automated workflows will be needed before moving to a clinical application.

Our approach includes first quantifying overall vorticity in the RH at peak diastole. Before vorticity can be compared between subjects, robust methods are needed for determining (1) the volume to integrate over; (2) a scaling to compensate for variance in patient size and heart rate; and (3) the time of peak diastole. Here, we present such methods, including a reliable method of estimating the timing of peak early diastole based on 4D Flow derived flowrates through the TV annulus and main pulmonary artery (MPA).

## 2. Materials and Methods

RH vorticity at peak diastole was compared between normal subjects and subjects with PAH and RVDD. Same-day 4D Flow and echocardiographic data was acquired for each subject. 4D Flow images were preprocessed to improve data quality, and qualitative and quantitative characteristics were assessed using ParaView visualization and quantification software (Kitware, Clifton Park, NY, USA) [21].

### 2.1. Data Acquisition

The study population consisted of 20 subjects with RVDD and 14 controls. All subjects gave their informed consent for inclusion before they participated in the study. The study was conducted in accordance with the Declaration of Helsinki, and the protocol was approved by the Institutional Review Board of National Jewish Health (Protocol #2808. Approved 1/23/14). RVDD subjects had RVDD per American Society of Echocardiography guidelines [22,23]. Echo-derived diastolic function was used as a surrogate for invasive RV end diastolic pressure measurements which may lead to over or under estimation of RV diastolic impairment. In order to exclude conditions that may confound RH flow patterns, subjects (both control and RVDD) with cardiomyopathy, coronary artery disease, significant valvular heart disease, or advanced liver disease were excluded from analysis. 

Two-dimensional and Doppler echocardiography were performed to obtain LV and RV diastolic parameters including MV and TV early (E) and late (A) filling peak velocities, E/A ratio, early diastolic deceleration time, and lateral and septal tricuspid and mitral early (e′) and late (a′) diastolic velocities. RVDD was defined as either stage I (TV E/A < 0.8, TV E/e′ > 6, and DT > 120 ms) or stage II (TV E/A = 0.8–2.1, E/e′ > 6, and DT > 120 ms) [23].

Time-resolved 3D phase-contrast cardiac MRI imaging was performed using a Siemens Avanto 1.5 T MRI scanner with the subject in the supine position. In-plane voxel dimensions were square and ranged from 1.98 mm to 2.60 mm depending on patient size, with a slice thickness of 3 mm for all patients. The field of view was rectangular with voxel volumes ranging from 11.75 to 20.35 mm^3^. Other scan parameters were α = 15°, TE/TR = 2.85/48.56 ms, venc = 100–150 cm/s and temporal resolution was 50 ms. An RF-spoiled gradient echo pulse sequence, prospective ECG gating, and respiratory navigators were used as described in [24]. Note that because of multi-cardiac cycle time averaging of the velocity data that occurs with MRI scans, stochastic properties are not present in the final velocity data which instead represents an ensemble average over multiple cardiac cycles.

In additional to phase-contrast images, steady state free precessing axial, short axis, 4-chamber, and 2-chamber 2D cine images were obtained for morphological and functional assessment. Short axis images with in-plane resolutions ranging from 1.09 to 1.56 mm, slice thickness of 6 mm, and voxel volumes ranging from 7.2 to 14.6 mm^3^ were obtained from the ventricular apex to beyond the tricuspid plane.

### 2.2. Preprocessing

Several 4D Flow data preprocessing tools and techniques were used in this study that have significant impact on the resulting data quality, including noise reduction and anti-aliasing algorithms.

The noise reduction algorithm was based on the tissue magnitude image method of Bock et al. [25] in which velocity data is set to zero in regions with low signal-to-noise. The tissue-contrast intensity threshold used for this study was set at 14 for all images which corresponds to approximately 3% of the maximum pixel intensity for the images. Velocity was set to zero in voxels corresponding to an intensity value of 14 or less in the spatially and temporally corresponding anatomical magnitude image. Visual analysis of noise-filtered images for a range of thresholds and subjects showed this value to be a good compromise between noise reduction and unwanted removal of velocity data.

**Figure 1:**
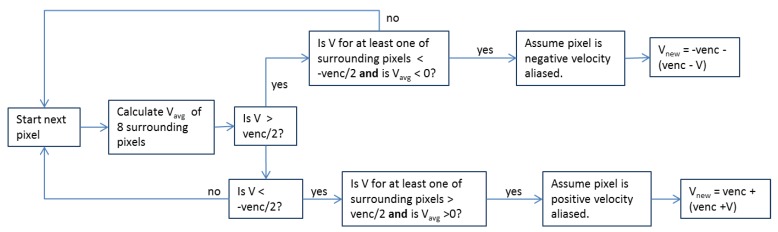
Anti-aliasing algorithm logic chart.

**Figure 2:**
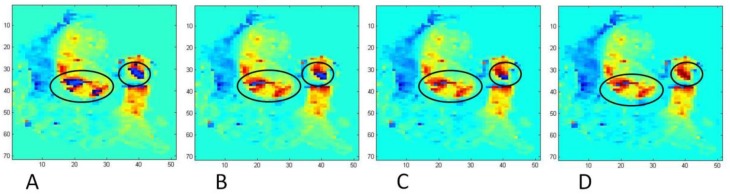
Results of the anti-aliasing algorithm on aliased image slices of the aorta and main pulmonary artery (MPA) demonstrating the first, second, third, and fifth iterations of the algorithm on a heavily aliased velocity image. Initially aliased areas are circled in black. No further aliasing is identified by the algorithm beyond the fifth iteration (panel D) and the code terminates, leaving panel D as the final corrected image.

Venc was initially set to 100 cm/s for an initial round of subjects due to measurements of bulk velocities that were not predicted to exceed this value. However, after noting significant aliasing in these scans, particularly in the MPA and ascending aorta during peak systole and in the region of the TV in patients with TV regurgitation, venc was increased to 150 cm/s for the remainder of the subjects, although some aliasing still occurred. To correct velocity aliasing, an iterative anti-aliasing algorithm was developed similar to that of Axel and Morton [26]. Shown in Figure 1 is a logic chart for the anti-aliasing algorithm, and shown in Figure 2 is a representative result from several iterations of the algorithm on a heavily aliased image. The code is set to terminate after a maximum of 10 iterations.

### 2.3. Vorticity Calculation

Vorticity was calculated in ParaView using a first order bilinear interpolation scheme over the entire RH for all subjects at each time-step in the cardiac cycle. The volume integrated vorticity workflow involved preprocessing raw data as previously described, converting velocity data and cine images to Ensight and VTK formats respectively, visualizing velocity and vorticity vectors in ParaView, thresholding vorticity vectors, summing vorticity vector magnitudes within a rectangular prismatic region of interest (containing either RA, RV, or RA + RV volumes), and multiplying the resulting summation by voxel volume to get a volume integration.

**Figure 3:**
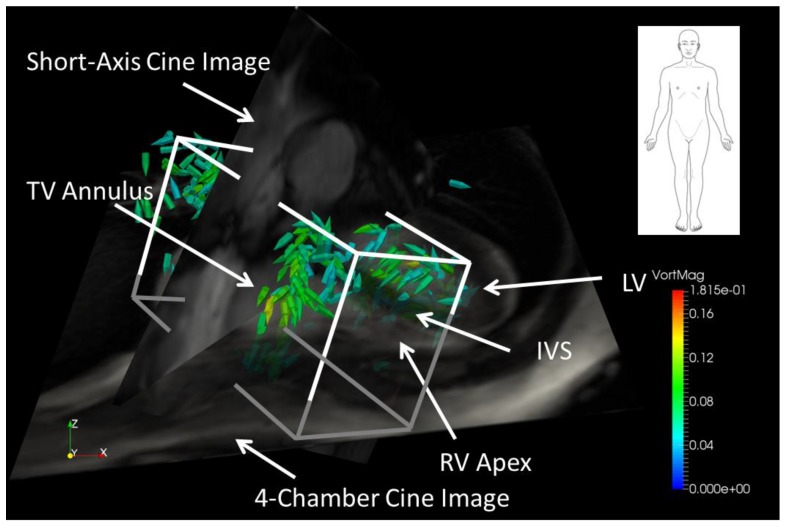
Vorticity vectors in a right ventricle (RV) volume during early diastole in a healthy subject. The cuboid encloses the RV volume used for volumetric vorticity integration. The red-scale 4-chamber view is semi-opaque and the short-axis view is fully opaque.

The combined RA + RV volume was manually isolated using a rectangular prism oriented along the RA/RV longitudinal axis using early diastolic vorticity vectors and 2D short-axis and 4-chamber MRI images as a visual guide (see Figure 3). The rectangular prism was oriented such that RH diastolic vorticity vectors were included in the volume, while any vorticity in the LV and ascending aorta was excluded. A subjective judgment was often made regarding how much of the inferior and superior vena cava to include in the RA—which may have an effect on the results, particularly in patients with diastolic backflow in the vena cava for which significant diastolic vorticity was present in the veins. However, an interobserver variability study, described elsewhere in this paper, indicates that the effect is small. In order to separate the RV from the RA, the early diastolic 4-chamber tissue contrast image was used to assist in locating the tricuspid plane at peak diastole.

### 2.4. Estimation of Cardiac Event Timing

The diastolic phase of the cardiac cycle is split into an early diastolic phase (E-wave) during which blood is drawn into the ventricle via ventricular relaxation, and late diastole (A-wave) in which atrial contraction forces additional blood into the ventricle. The timing of peak E-wave in the RH—defined here as the moment of greatest time rate of ventricular volume change during early diastole—was estimated using the peak of the tricuspid flow rate time series (described below). When a distinct E-wave peak was less apparent (generally in RVDD subjects), curves of the time derivative of LV volume were used in addition to TV flowrate to estimate peak early diastolic timing in the RV. The length of time between E- and A-waves was determined from the LV volume curves and was then subtracted from the A-wave TV flow peak time to yield the RV E-wave time for vorticity analysis. In addition, flowrates through the MPA were calculated, which, when combined with the TV flowrates, were used for comparison between right and left heart events.

**Figure 4:**
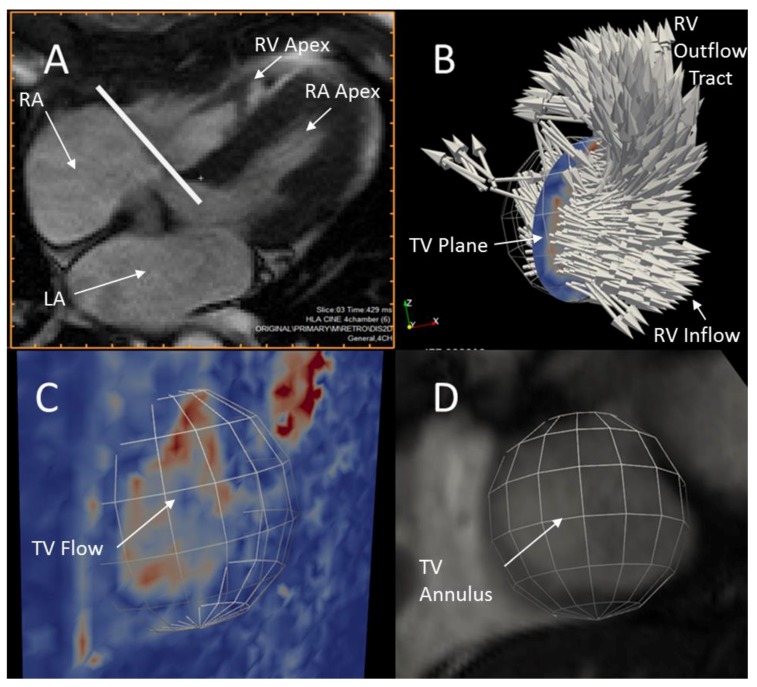
Available data used to locate the diastolic tricuspid valve (TV) plane and circular area. (**A**) 4-chamber cine tissue contrast image at early diastole with a line denoting the TV plane; (**B**) Velocity vectors at early diastole in the region of the tricuspid valve showing the disk location; (**C**) a plane colored by velocity magnitude at early diastole showing the spherical clip used to produce the disk; (**D**) A short-axis tissue contrast cine image showing the TV blood pool at early diastole.

Flowrates through the TV and MPA were calculated using 4D Flow velocity data and ParaView flow visualization software. The TV plane perpendicular to the direction of bulk blood flow was initially located visually using a cine animation of a mid-TV 4-chamber MRI tissue contrast slice at the approximate start of diastole as shown in Figure 4a. Figure 4b shows a spherical region of interest (“clip” in ParaView) combined with an interpolated slice of blood velocity data co-located with the TV plane to produce a disk of velocity data approximating the area of flow through the TV. The size and location of the disk was further refined by visually comparing it to early diastolic velocity magnitude images in the TV plane, the full field of 3D velocity vectors, and high-resolution short-axis tissue contrast images during all early diastolic time-steps (Figure 4c,d). The area-integrated normal flow through the disk was then calculated for each time-step using the ParaView Surface Flow filter and the resulting TV flowrate time-series was exported from ParaView. Due to movement of the tricuspid annulus during diastole, this method is only accurate at the time-step for which the disk position is optimized.

**Figure 5:**
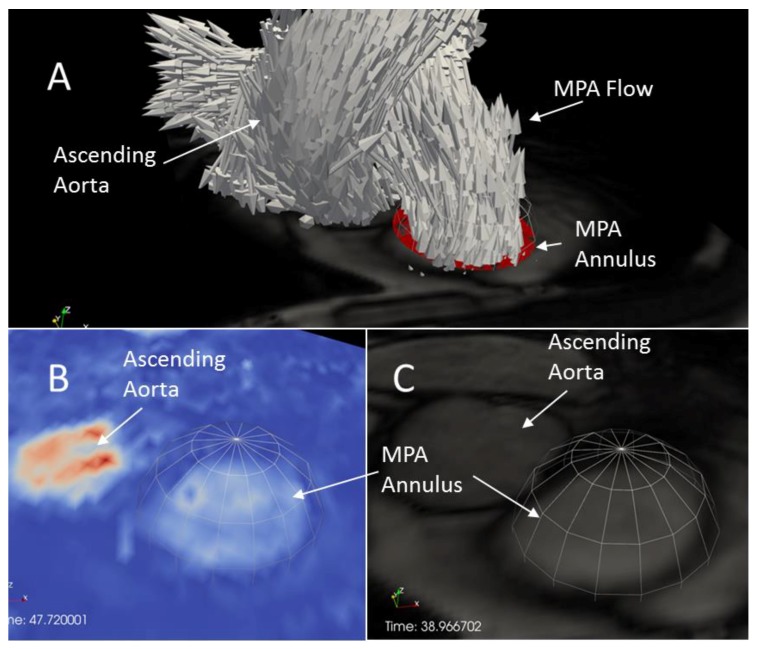
Data used in locating a disk in the MPA for MPA flowrate calculations. (**A**) An axial tissue contrast cine image with velocity vector glyphs and the disk colored in red during systole. Note the proximity of MPA and aortic vertical flow; (**B**) A slice of velocity data colored by velocity magnitude showing the spherical clip; (**C**) An axial tissue contrast cine image showing the spherical clip.

A plane approximately perpendicular to the MPA was located for each subject in a region between the pulmonary valve and the left/right pulmonary artery split using axial cine images. An initial location was found in which spatial separation between the MPA and aorta during systole was large enough to prevent aortic flow from contaminating the MPA velocity data. A method similar to that described above for TV flow was then used to refine the size and location of a disk over which normal velocity was integrated to produce a time series of MPA flowrate (See Figure 5). Because spatial mismatch can occur between 4D Flow and cine data, final location of the disks for TV and MPA flowrate calculation was performed using 4D Flow velocity data rather than cine data.

The LV volume as a function of time was determined from short-axis cine data. Ideally, RV volume would be used, but due to the complexity of RH geometry, automatic segmentation schemes are still in development and require high computational times relative to automatic LV segmentation [27]. The LV endocardium of each subject was semi-automatically segmented at each short-axis cine image time-step using the research version of Medviso’s Segment software, v1.9 R3763 (Medviso AB, Lund, Scania, Sweden) [28]. Each segmentation boundary curve was visually inspected for accuracy, and in rare cases in which the curve differed considerably from the visible endocardium, the curves were manually corrected in Segment. Segmentation curves were converted to LV volume curves within Segment and exported. The resulting LV volume time-series were exported for each subject and the central difference time derivative of volume for each subject was calculated at all time-steps. Forward difference and backward difference was used for the first and last time-steps respectively.

Isolating the RA, RV, or RA + RV volumes using a rectangular prism permits the undesirable influence of contributions from small regions of velocity that may lie outside the physical boundaries of the RH chambers. These velocities may be due to blood vessels, velocity noise in tissue regions, or tissue motion. Both these velocities and associated vorticity levels were low, so in order to reduce the sensitivity of the results on the subjective placement of the rectangular prismatic region of interest, small vorticity vectors were removed from the region of interest (ROI) prior to volume integration by setting all vorticity magnitudes below a threshold value to zero. This resulted in integration of the large vorticity vectors only, and a vorticity-free buffer region near the prismatic surface walls. Thus, small changes in surface placement have minimal effect on the integration, and numerical problems with partial cell integrations are avoided. Vorticity thresholding involved deciding on a vorticity magnitude threshold level for a single healthy subject, and then scaling that value for the remainder of the subjects using a cardiac flow parameter. Cardiac index (CI) was ultimately used as a vorticity threshold scaling parameter due to its incorporation of several parameters including heart rate, stroke volume, and body surface area. By scaling the threshold value to CI, the effect of body size and the flowrate of the heart are accounted for, resulting in a larger inter-subject effect due to changes in flow structures. In addition to the aforementioned CI scaled thresholding, vorticity was also thresholded at constant levels across all subjects in order to determine the impact of the choice of scaling parameter.

The resulting thresholded vorticity magnitude element summations were multiplied by voxel volume to get the numerical spatial integration of vorticity. Integrated vorticity was then scaled by CI to again reduce the dependency of the results on body mass, heart size, and heart rate; factors that are expected to influence overall vorticity. The resulting scaled and spatially integrated vorticity was then used as our metric of interest.

**Figure 6:**
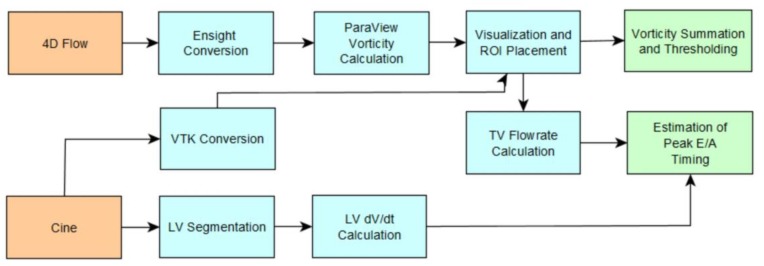
Process flowchart of postprocessing methodology using 4D Flow and Cine data to calculate spatially integrated vorticity and peak ealy (E) and late (A) wave timing.

RH vorticity at peak E-wave was calculated by two trained analysts using the method previously described for an initial 23-subject cohort consisting of 13 RVDD patients and 10 controls. The concordance correlation coefficient was calculated between the two analysts’ results for constant (unscaled) threshold levels ranging from 0.005 to 0.100 s^−1^ in 0.005 s^−1^ increments in order to examine the effect of threshold level on interobserver reliability. A flow chart of postprocessing steps is shown in Figure 6.

## 3. Results

Vorticity and peak A and E wave diastolic time were calculated for 14 controls and 20 RVDD patients. Control and RVDD characteristics are shown in Table 1.

### 3.1. Timing of Cardiac Events

Several important characteristics of cardiac MRI confound the ability to use 4D Flow and high-resolution cine data to approximate the timing of cardiac events. The first is the significant change that can occur in a subject’s heart rate during the MRI exam. For example, during the 4D Flow sequence, a heart rate range, or interval, is set within which an acquisition is accepted. However, this may vary considerably from the acceptable heart rate interval for cine sequences leading to difficulties comparing 4D Flow defined RH events and cine (i.e., automatic LV segmentation) defined left heart events. The second difficulty was that the 4D Flow sequence is prospectively gated (acquisitions are timed prospectively from a QRS complex trigger) while the cine data is retrospectively gated. The third difficulty is the low temporal resolution of the 4D Flow data; here, 50 ms was used as a compromise between data quality and total MRI exam time.

For an individual subject, nominal interval (the average duration of cardiac intervals for which images are accepted) was found to vary significantly within a single cine image orientation and between cine images and 4D Flow. The largest range for nominal interval within a single cine series was 38.3% of the mean—occurring for a short-axis image series in which nominal interval ranged from 850 to 1199 ms with a mean of 910 ms. The average range for all cine images was 8.88% of their respective means. The largest magnitude of percentage difference between average short axis cine nominal interval and corresponding 4D Flow nominal interval was 10.7% (4D Flow NI = 994 ms, cine NI = 627 ms) while the absolute minimum was −7.08% (4D Flow NI = 1057 ms, cine NI = 1138 ms).

**Figure 7:**
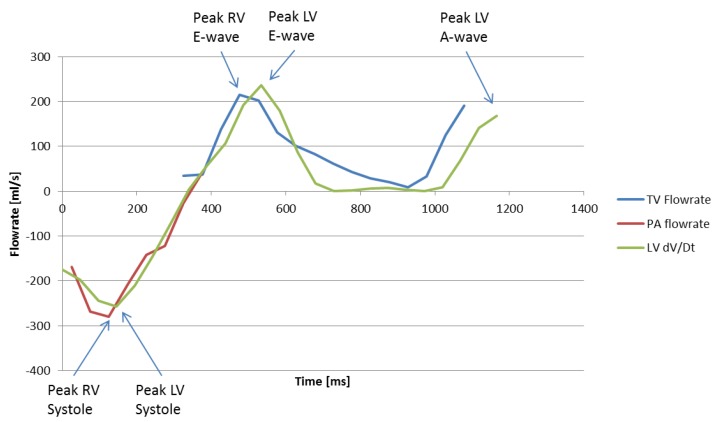
TV and MPA flowrates and time derivative of LV volume for a normal subject.

Despite these limitations, the 4D Flow TV and MPA flowrate method was found to be an accurate but labor-intensive method for estimating peak systolic and early diastolic timing in the left and right ventricles. Figure 7 shows flowrates through the TV and MPA as well as the time derivative of LV volume (from cine data) at discrete time-steps in the cardiac cycle of a normal subject. Peak LV and RV systole and E-wave are clearly visible on the plot. However, due to prospective 4D Flow gating, the TV velocity data is potentially truncated at the end of the cardiac cycle and it becomes ambiguous whether the last time-step represents true peak RV A-wave. Note also from the figure that the major cardiac events between the left and right heart correspond well, with the LH events slightly delayed roughly 20–50 ms. It is unclear whether this delay is due to variation in heart rate between 4D Flow (the data used for TV and MPA flowrate calculation) and cine (data used for LV volume calculation) data acquisition, or if the delay is caused by prospective gated 4D Flow versus retrospective gated cine acquisition techniques.

**Figure 8:**
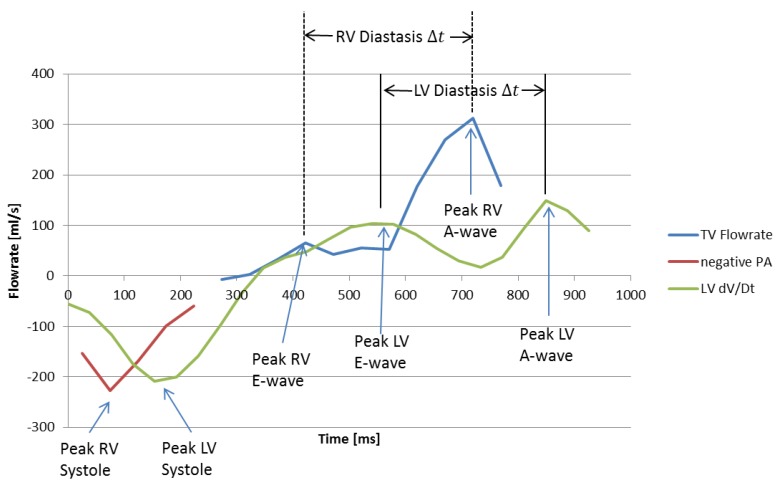
TV and MPA flowrates and time derivative of LV volume for a subject with RVDD. The RV E-wave peak at 410 ms is chosen by working backwards from the RV A-wave by a time equivalent to the LV diastasis ∆t.

Figure 8 shows RV flowrate and LV volume curves for a subject with Stage 1 RVDD. In contrast to normal RV filling patterns in which peak E velocity is greater than peak A velocity (see previous image for comparison), Stage 1 RVDD is characterized by A > E peak velocity. As expected, RV A-wave has become dominant over E-wave. Calculating peak E-wave requires examination of the corresponding LV volume curve in order to estimate a time difference between early and late diastole and then working backwards from LV late diastole. Although the E-wave peak is small, it can be seen that a peak does exist at the time that early diastole would be expected. Because changes in heart rate are largely driven by changes in diastolic duration [29], the significant delays in heart events between the left and right heart in this subject can be explained by a decreased HR during the cine portion of the scan used for LV dV/dT data (63.9 bpm, 929 ms cardiac cycle duration) versus the 4D Flow portion (67.3 bpm, 891 ms cardiac cycle duration) combined with a roughly 80 ms constant temporal offset resulting from prospective/retrospective gating differences in the scans.

### 3.2. Interobserver Reliability

**Figure 9:**
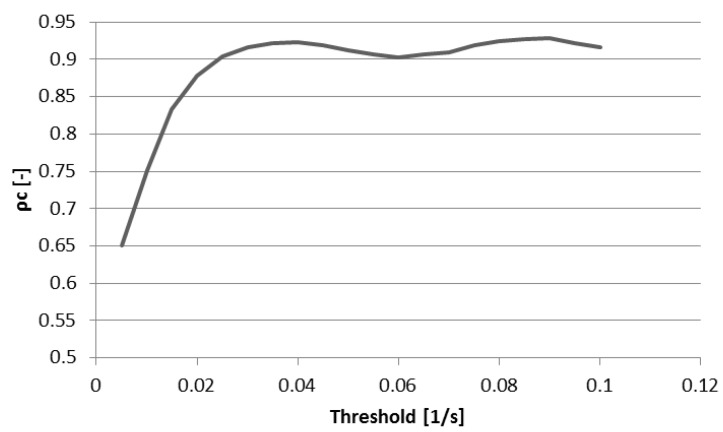
Concordance correlation coefficient of vorticity for two observers at multiple vorticity thresholds. Concordance increases with increasing vorticity threshold until a threshold of roughly 0.04 L/s.

**Figure 10:**
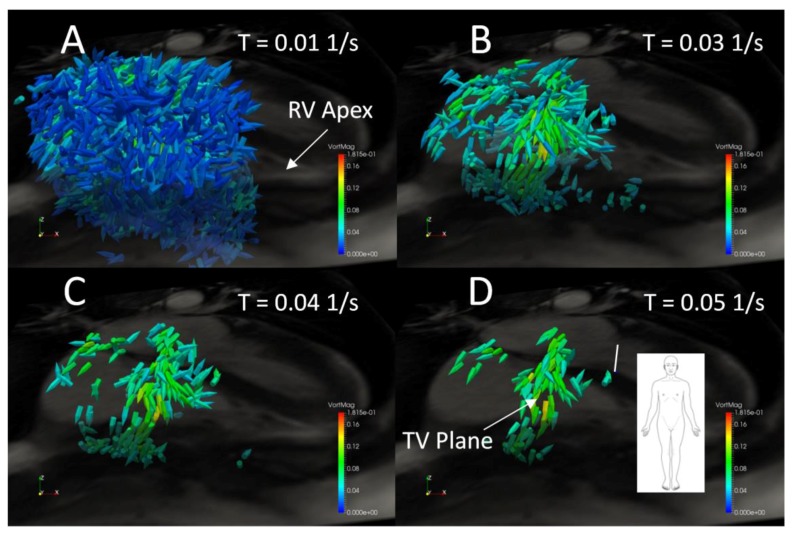
Right heart vorticity during peak early diastole for a normal subject thresholded at (**A**) 0.01 s^−1^, (**B**) 0.03 s^−1^, (**C**) 0.04 s^−1^, and (**D**) 0.05 s^−1^. Vector glyphs are colored by vorticity magnitude and a grayscale 4-Chamber cine image plane is shown for spatial reference.

Figure 9 shows concordance coefficient, ρ_c_, of vorticity for two observers over a range of vorticity thresholds. The coefficient exceeds 0.90 at a threshold of 0.025 s^−1^ and reaches a local peak of 0.92 with a threshold of 0.04 after which little is gained in interobserver reliability with increasing thresholds. Figure 10 shows the effect of increasing threshold on the vorticity vectors in the right heart of a normal subject. As the threshold reaches 0.04 s^−1^, a coherent ring of vorticity resolves in the middle of the TV constriction. At 0.05 s^−1^, gaps appear in the ring, indicating excessive thresholding and loss of data pertaining to the structure. Based on the threshold images and the concordance coefficient, it appears that 0.04 represents an approximate compromise between excessive data loss and interobserver reliability.

### 3.3. Peak E-Wave Vorticity

Peak early diastolic vorticity in the RV, RA, and RH volumes was integrated within a rectangular prism around the RH using several scaling and thresholding schemes as detailed in the methods section. T-tests were performed to evaluate differences in vorticity between the RVDD group and the normal group for all combinations of thresholding and CI scaling to investigate the effect of these methods on differences between the two groups and to elucidate the optimal technique for potential clinical RVDD diagnostic t-tests between the RVDD and normal groups.

Table 2 shows the results ordered in descending statistical significance for all scaling techniques for which the *p*-value is less than 0.05. Several trends are evident in the results. First, of the four scaling/thresholding schemes used (no scaling of integrated vorticity plus use of a constant value for thresholding, integrated vorticity scaled by CI, vorticity thresholded by CI scaled values, and a combination of both—None, SS, TS, and SS/TS respectively) only the SS and TS methods result in the detection of significant differences in the populations (*p* < 0.05). With a stricter significance definition, only the SS method resulted in significant differences (*p* < 0.025). Second, statistically significant (*p* < 0.05) differences between vorticity in the two populations is evident only in the RH and RA regions of interest and not in the RV. Third, 95% statistical significance occurs for a range of threshold values from 0.03 to 0.06, with more cases occurring with thresholds of 0.03–0.04 (0.03 N = 3, 0.04 N = 3, 0.05 N = 2, 0.06 N = 2). The highest statistical significant occurred with an RA ROI, a constant threshold of 0.04, and with integrated vorticity scaled by CI for which the *p*-value was 0.011.

## 4. Discussion

In this paper, we detail a method for quantitatively examining early diastolic vorticity in the RA, RV and RH regions of the human heart. In order to arrive at a statistical analysis of the difference in spatially integrated vorticity between an RVDD population and controls, several intermediate steps are involved including data preprocessing and quality control, analysis of right heart cardiac event timing, development of a methodical workflow for defining the regions of interest, and assessing interobserver reliability.

Ideally, the time of peak early diastolic flow through the tricuspid valve would be determined uniquely from a first peak in TV volume flowrate. This is possible in normal subjects, although it is time consuming due to the motion of the TV. In RVDD patients, where the E-wave peak is ambiguous, a hybrid method was developed. Fast and accurate automated LV segmentation allows determination of the LV volume, and thus detecting the peak of the time derivative of LV volume curves was found to be a practical method for estimating the timing of cardiac events in the normal left heart including peak systole, and peak early and late diastole. However, the LV volume curves do not provide directly transferrable times for the right heart flows due to (1) limited time resolution of the 4D flow data and (2) differences in heart rate between the cine scans (from which LV volume is derived) and the 4D flow measurements. As a compromise, the length of time between E- and A-waves was determined from the LV volume curves and was then subtracted from the A-wave TV flow peak to yield the E-wave time for vorticity analysis. Although this method is not ideal, it provides repeatable results for both normal and RVDD subjects.

Interobserver variability of vorticity integration was assessed for a 23-subject subset of the cohort using two trained analysts. Because we theorized that thresholding the vorticity vectors prior to spatial integration would both focus the analysis on larger coherent flow structures in the RH as well as reduce the dependency of the results on small differences in placement of the rectangular prismatic ROI, we calculated a concordance coefficient for several vorticity thresholds ranging from 0.005 to 0.01 s^−1^ (0.005 s^−1^ increments) and found that good agreement began with a threshold of roughly 0.04 s^−1^, above which gains in agreement were minimal. To elucidate the dependency of interobserver reliability on vorticity threshold, we examined images of early diastolic RH vorticity vectors for several threshold levels in a normal subject and observed that at a threshold of roughly 0.03–0.04 s^−1^ noise is reduced and flow structures gain visual coherence. 

Spatially integrated early diastolic RH vorticity was compared between the RVDD and control groups using four methods of threshold and integrated vorticity scaling. Statistically significant differences were found for three of the methods, and the greatest significances generally occurred in the RA using thresholds ranging from 0.03 to 0.06 s^−1^ and both CI scaled integrated vorticity (SS) and CI scaled thresholds (TS). RVDD subjects thus exhibited less total vorticity than the controls, after correcting for cardiac index differences. No significant differences were found between the two groups when vorticity was not scaled. Less significant differences were found between the two groups when both the SS and TS scaling methods were used in conjunction. It is theorized that this “double” scaling only serves to convolute the results while a single scaling elucidates differences in vorticity between RVDD and controls by reducing the dependence of vorticity values on heart rate and stroke volume. The highest significance, *p* = 0.011, occurred with a threshold of 0.04 s^−1^ and the SS scaling method.

## 5. Conclusions

The results of vorticity analysis, together with the interobserver variability analysis, indicate that assessing peak spatially integrated vorticity using a threshold of 0.04 s^−1^ and scaling the results by CI is a viable vorticity metric for the study of RVDD pathology as well a possible RVDD metric for further investigation for clinical use. This proof of concept study has shown that there are significant differences in 3D right heart flow characteristics between normal and RVDD subjects. Additionally, in this study, we have developed and described tools for examination of the basic fluid dynamics of the right heart which may then lead to a selection of a metric for disease progression. These tools and techniques provide a foundation for more sophisticated automated coherent structure analysis techniques including Q-criterion, Lambda2, helical decomposition, and Lagrangian coherent structures.

## Figures and Tables

**Table 1:** Control and right ventricular diastolic dysfunction (RVDD) cohort characteristics. Values are mean ± SE. BMI, body mass index; CO, cardiac output; CI, cardiac index; E/A, Tricuspid early and late velocity ratio; E/e’, Early fluid to lateral tricuspid velocity ratio; ERVEDV, right ventricular end diastolic volume; RVESV, right ventricular end systolic volume; RVEF, right ventricular ejection fraction.

		Control (*n* = 14)	RVDD (*n* = 20)
BMI	[kg/m^2^]	25 ± 2	28 ± 1
CO	[L/min]	4.3 ± 0.3	4.4 ± 0.3
CI	[L/min/m^2^]	2.4 ± 0.2	2.5 ± 0.2
E/A	[-]	21.8 ± 0.2	1.1 ± 0.1
E/e′	[-]	3.4 ± 0.3	6.4 ± 0.6
RVEDV	[mL]	138 ± 9	181 ± 17
RVESV	[mL]	69 ± 6	123 ± 17
RVEF	[%]	50 ± 2	35 ± 3

**Table 2:** Results for spatially integrated peak early diastolic vorticity in three regions of interest for several scaling methods. SS = integrated vorticity scaled by cardiac index, TS = vorticity thresholded by cardiac index. Only results showing a *p*-value of less than 0.05 are shown. *P*-values increase from left to right.

	*p* < 0.05									
p	0.011	0.012	0.015	0.024	0.025	0.029	0.034	0.045	0.047	0.048
ROI	RA	RA	RA	RA	RA	RA	RH	RA	RA	RH
Scaling/Thgreshold Method	SS	SS	SS	SS	TS	TS	TS	SS	TS	SS
Thgreshold [L/s]	0.04	0.05	0.06	0.03	0.04	0.03	0.05	0.04	0.06	0.03
Normal Mean Vorticity [L/s or m^2^/60]	709	425	255	1121	1826	2685	1184	1353	750	2051
Normal Standard Deviation	325	219	145	466	886	1026	712	518	531	707
RVDD Mean Vorticity [L/s or m^2^/60]	408	222	127	749	1109	1870	653	887	387	1450
RVDD Standard Deviation	303	208	136	417	834	998	632	785	449	995
